# Probiotic *Lactobacillus* spp. Act Against *Helicobacter pylori*-induced Inflammation

**DOI:** 10.3390/jcm8010090

**Published:** 2019-01-14

**Authors:** Yi-Hsing Chen, Wan-Hua Tsai, Hui-Yu Wu, Chun-Ya Chen, Wen-Ling Yeh, Ya-Hui Chen, Hui-Ying Hsu, Wei-Wei Chen, Yu-Wen Chen, Wen-Wei Chang, Tzu-Lung Lin, Hsin-Chih Lai, Yu-Hsin Lin, Chih-Ho Lai

**Affiliations:** 1 Research and Development Department, GenMont Biotech Incorporation, Tainan 74144, Taiwan; ethan@genmont.com.tw (Y.-H.C.); twh@genmont.com.tw (W.-H.T.); wentz@genmont.com.tw (W.-L.Y.); yahui@genmont.com.tw (Y.-H.C.); 2 Department of Microbiology and Immunology, Graduate Institute of Biomedical Sciences, Chang Gung University, Taoyuan 33302, Taiwan; winney0614@gmail.com (H.-Y.W.); magicgirl0203@gmail.com (C.-Y.C.); ttoday3268@gmail.com (Y.-W.C.); 3 Department of Laboratory Medicine, Taichung Veterans General Hospital Chiayi Branch, Chiayi 60090, Taiwan; 4 Department of Medical Research, Graduate Institute of Biomedical Sciences, China Medical University and Hospital, Taichung 40202, Taiwan; tennainging@yahoo.com.tw (H.-Y.H.); x790107@gmail.com (W.-W.C.); 5 Department of Biomedical Sciences, Department of Medical Research, Chung Shan Medical University and Hospital, Taichung 40201, Taiwan; changwenwei@gmail.com; 6 Department of Medical Biotechnology and Laboratory Science, Microbiota Research Center, Chang Gung University, Gueishan 33302, Taiwan; f87445101@ntu.edu.tw (T.-L.L.); hclai@mail.cgu.edu.tw (H.-C.L.); 7 Molecular Infectious Disease Research Center, Department of Pediatrics, Department of Laboratory Medicine, Chang Gung Memorial Hospital, Linkou 33305, Taiwan; 8 Department of Pharmacy, National Yan-Ming University, Taipei 11221, Taiwan; 9 Department of Nursing, Asia University, Taichung 41354, Taiwan

**Keywords:** *Helicobacter pylori*, *Lactobacillus*, probiotic, inflammation

## Abstract

The bacterial species, *Helicobacter pylori*, is associated with several gastrointestinal diseases, and poses serious health threats owing to its resistance to antibiotics. *Lactobacillus* spp., on the other hand, possess probiotic activities that have beneficial effects in humans. However, the mechanisms by which *Lactobacillus* spp. harbor favorable functions and act against *H. pylori* infection remain to be explored. The aim of this study was to investigate the ability of bacterial strains, *Lactobacillus rhamnosus* and *Lactobacillus acidophilus*, termed GMNL-74 and GMNL-185, respectively, to inhibit *H. pylori* growth and inflammation. Our results showed that GMNL-74 and GMNL-185 possess potent antimicrobial activity against multidrug resistant (MDR)-*H. pylori*. In addition, an in vitro cell-based model revealed that the inhibition of *H. pylori* adhesion and invasion of gastric epithelial cells and interleukin-8 production were significantly decreased by treatment with both the *Lactobacillus* strains. In vivo studies demonstrated that colonization of *H. pylori* and induced inflammation in the mouse stomach were also alleviated by these *Lactobacillus* strains. Furthermore, the abundance of beneficial gut bacteria, including *Bifidobacterium* spp. and *Akkermansia muciniphilia*, were significantly increased in *H. pylori*-infected mice treated with GMNL-74 and GMNL-185. These results demonstrate that *Lactobacillus* spp. ameliorate *H. pylori*-induced inflammation and supports beneficial gut specific bacteria that act against *H. pylori* infection.

## 1. Introduction

*Helicobacter pylori* is a gram-negative, microaerophilic, spiral-shaped bacterium that colonizes the stomach mucosa and causes gastrointestinal diseases [1]. Epidemiological analyses revealed that nearly one-third of the population of North Europe and North America was infected with *H. pylori*, whereas in Southern and Eastern Europe, South America, and Asia, more than half the population suffered from *H. pylori* infection [2]. This infection is associated with atrophic gastritis [3] and gastric cancer [4,5], thus suggesting that eradication of *H. pylori* can reduce the incidence of such diseases.

Two major virulent factors of *H. pylori* are reported to induce pathogenesis: Cytotoxin associated gene A (CagA) and vacuolating cytotoxin A (VacA). CagA, encoded by *cag*-pathogenicity island (PAI), is delivered into host cells via the Type IV secretion system (TFSS) [6,7]. Translocation of CagA in host cells induces nuclear factor-kappa B (NF-κB) activation and production of interleukin 8 (IL-8), a chemokine responsible for neutrophil activation [8]. Moreover, CagA-positive *H. pylori* strains are known to be more virulent than CagA-negative strains [9]. In addition, VacA, secreted from *H. pylori* [10], disrupts host mitochondrial functions and induces apoptosis [11,12] in the host cells. 

Epidemiological studies show a close association between the prevalence of *H. pylori* and dyspeptic symptoms. *H. pylori* eradication cures gastritis and alters the complication or recurrence of gastrointestinal diseases, indicating that *H. pylori* is an infectious disease in humans [13]. A common treatment for *H. pylori* infection is triple therapy, which combines proton pump inhibitors (PPI), clarithromycin, metronidazole, or amoxicillin [14,15]. Recently, Maastricht IV guidelines indicated that in areas of high dual clarithromycin and metronidazole resistance, bismuth-based quadruple therapy is recommended as a first-line treatment [13]. However, failure with such a treatment has been seen to increase year by year, one of the reasons being bacterial resistance to antibiotics [16]. Therefore, alternative approaches are urgently required to combat *H. pylori* infection. 

Probiotics are live microorganisms that provide potential health benefits to humans, such as improved immune responses [17,18,19] and defense against pathogenic bacteria [20,21]. A majority of probiotics are of the genera, *Lactobacillus* and *Bifidobacterium* [22]. Compelling evidence suggests that most of the *Lactobacillus* and *Bifidobacterium* strains possess properties of acid tolerance and antimicrobial activity [23,24,25]. Due to its property of acid resistance, *Lactobacillus* could potentially act as delivery vectors for medical therapies, such as vaccines [26,27]. In addition, *Lactobacillus* is demonstrated to have antagonistic effects toward *H. pylori* in vitro [28]. However, the mechanisms by which *Lactobacillus* inhibit *H. pylori* require further investigation. In this study, we aim to investigate the ability of *Lactobacillus* strains possessing potent anti-*H. pylori* activity and the attenuation of inflammatory responses. Our results show that these *Lactobacillus* strains contain the activities to curb the growth of multidrug-resistant (MDR) *H. pylori* and ameliorate *H. pylori*-induced inflammation by altering gut specific bacteria in a murine model. 

## 2. Materials and Methods

### 2.1. Chemicals and Antibodies

Antibodies against to *H. pylori* CagA and phosphotyrosine (4G10) of CagA were purchased from Santa Cruz Biotechnology (Santa Cruz, CA, USA) and Millipore (Temecula, CA, USA), respectively. Lipofectamine 2000 were purchased from Invitrogen (Carlsbad, CA, USA). Antibodies specific to cyclooxygenase-2 (COX-2) and Tumor necrosis factor (TNF)-α were purchased from Sigma-Aldrich (St. Louis, MO, USA). The NF-κB-luc promoter construct was kindly provided by Dr. Chih-Hsin Tang (China Medical University, Taichung, Taiwan) [29]. All other chemical and reagents were purchased either from Sigma–Aldrich or Merck (Whitehouse Station, NJ, USA). 

### 2.2. Cell Culture

AGS cells (human gastric adenocarcinoma epithelial cells, ATCC CRL 1739, Bioresource Collection and Research Center, Hsinchu, Taiwan) were maintained in F12 medium (Invitrogen, Carlsbad, CA, USA) with 10% de-complement FBS (HyClone, Logan, UT, USA) in a 5% CO_2_ humidified atmosphere at 37 °C as described previously [30]. 

### 2.3. Identification of Lactobacillus Strains that Possess Probiotic Activities

A total of 226 *Lactobacillus* strains from the Probiotic Bank (GenMont Biotech, Tainan, Taiwan) were initially screened to study inhibition of adhesion and invasion of *H. pylori* gastric epithelial cells. Two probiotics, GMNL-74 and GMNL-185, possessing the most potent activities, were identified to further examine their strain specificity. Species and strain specificity of GMNL-74 and GMNL-185 were determined by 16S rDNA sequencing. Randomly amplified polymorphic DNA (RAPD) profiles were generated by amplifying DNA of GMNL-74 and GMNL-185 with the primer, LacP2 (5’-ATGTAACGCC-3’), by PCR, followed by DNA agarose gel analysis. The ability of these *Lactobacillus* strains to ferment carbohydrates was determined using the API 50 CHL system (bioMérieux, Durham, NC, USA) to identify *Lactobacillus* strains. The carbohydrate utilization patterns of GMNL-74 or GMNL-185 were then analyzed. Microbial identification kits supplied by bioMérieux were used to identify GMNL-74 and GMNL-185 as *L. rhamnosus* and *L. acidophilus,* respectively. The data was analyzed using the bioMérieux website (https://apiweb.biomerieux.com/jsp/ident/index.jsp). 

### 2.4. Bacterial Strains and Culture

*H. pylori* 26695 (ATCC 700392) was used as a reference strain as described previously [31]. Multidrug resistant (MDR) *H. pylori* strains (v633 and v1354) were clinical isolates, which were characterized as resistant to both metronidazole and clarithromycin [32]. All *H. pylori* strains were routinely cultured on Brucella blood agar plates (Becton Dickinson, Franklin Lakes, NJ, USA) containing 10% sheep blood under 5% CO_2_ and 10% O_2_ conditions at 37°C for 48 h. *Lactobacillus rhamnosus* GMNL-74 (GM-020^®^, CCTCC M 203098, BCRC 910236) and *Lactobacillus acidophilus* GMNL-185 (CCTCC M 2017764, BCRC 910774) were cultured on *Lactobacilli* MRS agar plates (Becton Dickinson) at 37 °C for 24 h as described previously [33]. To activate *Lactobacillus* spp., the bacterial strains were cultured in MRS broth at 37 °C for 24 h and then subjected to each experimental study. 

### 2.5. Determination of Anti-H. pylori Activity by Lactobacillus Strains

Anti-*H. pylori* activities of *Lactobacillus* spp. were determined by the disc agar diffusion method as described previously [31]. Briefly, reference or MDR *H. pylori* suspension (1×10^8^ colony forming units (CFUs)/mL) was spread on Mueller Hinton agar plates (BBL, Sparks, Maryland, USA) containing 10% sheep blood. The overnight culture of *Lactobacillus* strains (GMNL-74 and GMNL-185) were diluted in MRS broth (1:100) and incubated at 37°C for 24 h. The bacterial culture was adjusted to OD_600_ = 1 (ca. 7.6 × 10^6^ CFU/mL) and added to the paper discs. The plates were cultured in microaerophilic condition for 72 h and the inhibition zone was determined in diameter.

### 2.6. Assays for H. pylori Adhesion and Invasion to AGS Cells

Analyses of anti-adhesion and anti-invasion of *H. pylori* to AGS cells by *Lactobacillus* spp. were performed as previously reported [34]. Briefly, the live or heat-inactivated *Lactobacillus* strains suspension were added directly to the cell culture at an multiplicity of infection (MOI) of 100 for 10 min prior to inoculation with *H. pylori* 26695 (MOI = 100) to cells and incubation for 6 h. To determine the number of cell-adhesion bacteria, the infected cells were washed three times to remove unbound *H. pylori* and lysed with distilled water for 10 min. The lysates were then diluted in PBS and plated onto Brucella blood agar plates. After being cultured for 3–4 days, the viable CFUs were counted. To determine the number of intracellular *H. pylori*, the infected cells were washed three times in PBS followed by incubation with 100 μg/mL gentamicin (Sigma–Aldrich, Whitehouse Station, NJ, USA) for 1.5 h at 37 °C to remove extracellular bacteria. The following protocol was the same as above to obtain the viable CFUs. Results were determined by three independent experiments. The controls, containing *H. pylori* infected AGS cells, without *Lactobacillus* strains, were used to define as 100% adhesion or invasion. Results were expressed as the percentage of relative inhibition of *H. pylori* adhesion or invasion, by comparison with the controls, respectively.

### 2.7. NF-κB Reporter Luciferase Assay

AGS cells were cultured in a 12-well plate to be 90% confluent and transfected with NF-κB-luc reporter plasmid using Lipofectamine 2000 (Invitrogen) as described previously [35]. Briefly, AGS cells were treated with *Lactobacillus* strains (GMNL-74 or GMNL-185) followed by infection with *H. pylori* at MOI of 100 for 6 h. The transfected cells were lysed, and luciferase assays were performed with the Dual-Luciferase Reporter Assay System (Promega, Madison, MA, USA) using a microplate luminometer (Biotek, Winooski, VT, USA). Luciferase activity was normalized for transfection efficiency by the co-transfected with β-galactosidase expression vector (Promega) [30].

### 2.8. Measurement of Interleukin (IL)-8 Production

AGS cells were treated with *Lactobacillus* strains (GMNL-74 or GMNL-185), the treated cells were then infected with *H. pylori* at MOI of 1:100 for 6 h. The supernatants from the cell culture were collected and the levels of IL-8 in supernatants were determined by using a sandwich enzyme-linked immunosorbent assay (ELISA) kit (R&D Systems, Minneapolis, MN, USA), according to the manufacturer’s protocol [36]. 

### 2.9. H. pylori CagA Translocation and Phosphorylation Assay

Analysis of the levels of CagA translocation and phosphorylation were performed as described previously [37]. Briefly, AGS cells were treated with *Lactobacillus* strains (GMNL-74 or GMNL-185), and then infected with *H. pylori* at MOI of 1:100 for 6 h. The cell lysates were prepared and subjected to 6.5% SDS-PAGE then transferred onto polyvinylidene difluoride (PVDF) membrane (Pall, East Hills, NY, USA) for Western blot analysis. CagA and phospho-CagA were probed with mouse anti-CagA antibody (Santa Cruz Biotechnology); anti-phosphotyrosine antibody (Millipore). The proteins of interest were visualized using enhanced chemiluminescence reagents (GE Healthcare, Buckinghamshire, UK) and were detected by exposure to X-ray film (Kodak, Boca Raton, FL, USA). 

### 2.10. Animal Study and Immunohistochemistry Analysis

Male BALB/c mice that were 6-weeks old were obtained from the National Laboratory Animal Center of Taiwan. The mice were treated in accordance with the Animal Care and Use Guidelines for Chang Gung University under a protocol approved by the Institutional Animal Care Use Committee (IACUC Approval No.: CGU16-004). The experimental protocol was performed from June 1, 2016 to May 31, 2017, in accordance with the guidelines. Mice were divided into four groups, including untreated control (*n* = 6), *H. pylori* alone (*n* = 4), treatment with GMNL-74 and *H. pylori* infection (*n* = 4), or treatment with GMNL-185 and *H. pylori* infection (*n* = 5). Mice were fed with *Lactobacillus* spp. (8.2 × 10^6^/day) for a total of 24 days starting from 8 weeks of age. For experimental groups, mice were inoculated with *H. pylori* (1 × 10^8^) by intragastric gavage for a total of six administrations (at days 8, 10, 12, 14, 16, and 18). On the 25th day after treatment, mice were euthanized and the gastric tissues were prepared for hematoxylin-eosin (H&E) and immunohistochemistry (IHC) staining as described previously [38]. Briefly, tissue sections from the mouse stomach were de-paraffinized, rehydrated, and blocked with 3% bovine serum albumin. The samples were stained with antibodies specific to COX-2 and TNF-α for 24 h at 4 °C, and then probed with a horseradish peroxidase-labeled goat anti-rabbit secondary antibody (Epitomics, Burlingame, CA, USA) and developed with an ABC kit (Vector Laboratories, Burlingame, CA, USA). To assess the *H. pylori* infection in the stomach of the mice, gastric tissues were prepared for determining the urease by the CLO test (Kimberly Clark, Draper, UT, USA), which is the most widely used rapid urease test for the diagnosis of *H. pylori* infection [39]. Briefly, a section of gastric tissue was inserted into a microtube and kept at room temperature. After 3 h of incubation, the colonization of *H. pylori* was assessed by evaluating the change in color as described previously [40].

### 2.11. Stool Collection

Mouse stool (180–220 mg) were collected on Day 0 and Day 24 and transferred to a 2 mL tube containing RNAlater and stored at −80°C. All stool samples were extracted by following the protocol of the QIAamp DNA stool Mini Kit (Qiagen, Germany). The concentration of DNA was determined by Thermo Scientific NanoDrop Lite (Thermo Scientific, Wilmington, DE, USA). 

### 2.12. Analysis of Gut Specific Bacteria using Quantitative Real-time PCR (qRT-PCR)

The oligonucleotide primers for qRT-PCR quantification of total bacteria, bacterial phylum (*Bacteroidetes*, *Actinobacteria*, *Firmicutes*, *Proteobacteria*, *Fusobacteria*), genus (*Bifidobacterium*, *Prevotella*, *Clostridium* cluster I, *Enterococcus*, *Lactobacillus*), and species (*A. muciniphilia*, *S. aureus* and *E. coli*) are shown in Table 1. Each reaction included 5 µl SYBR Green Master Mix (Rotor-Gene^R^ SYBR^R^ Green, Qiagen, Germantown, MD, USA), primer mixtures (0.66 µM), and stool DNA samples (1 ng/µL). The reaction conditions for the amplification of DNA were 95 °C for 10 min, followed by 40 cycles at 95 °C for 10 s and 60 °C for 1 min each (Rotor-Gene-Q^R^, Qiagen, Germantown, MD, USA). Real-time PCR data for each bacteria quantity were calculated by relative calculation using the delta delta Ct method, (2^−ΔΔCt^). The method was used to calculate the relative abundance (fold changes) of each bacterial group. ΔCt was calculated as the difference between the Ct value with the primers to a specific group of bacteria and the Ct value with the primers to total bacteria. ΔΔCt is defined as the difference between the ΔCt value of each treatment and the ΔCt value of T0. The values derived from the 2^−ΔΔCt^ method show the fold changes of bacterial abundance in a treated sample relative to those of the T0 sample. The 2^−ΔΔCt^ value of T0 samples is equal to 1.

### 2.13. Statistical Analysis

The data are presented as mean ± standard deviation of independent triplicate experiments. The Student’s *t*-test was used to calculate the statistical significance of the experimental results between two groups. In the gut specific bacteria assay, a statistically significant difference in those groups was analyzed by using the Kruskal-Wallis test with post hoc test by Dunn test. A *p*-value less than 0.05 was considered significant. 

## 3. Results

### 3.1. Lactobacillus spp. Inhibits MDR-H. Pylori Growth 

A total of 226 *Lactobacillus* strains from the Probiotic Bank (GenMont Biotech, Tainan, Taiwan) were initially screened to study the binding of *H. pylori* to gastric epithelial cells. Out of these strains, two probiotics, GMNL-74 and GMNL-185, which possessed the most potent activities, were selected to further examine their strain specificity (Figure S1). In addition, *Lactobacillus* culture broth, antimicrobial agents, and two *Lactobacillus* strains (GMNL-229/GMNL-814) with less potent activity were used as control groups. Based on 16S rDNA sequence analysis, RAPD profiles (Figure S2), and API 50CHL (Table S1), GMNL-74 and GMNL-185 were identified as *Lactobacillus rhamnosus* and *Lactobacillus acidophilus*, respectively. We further evaluated the inhibitory activity of these *Lactobacillus* strains against *H. pylori* growth. By using the agar disk diffusion method, GMNL-74 and GMNL-185 exhibited an inhibitory effect against *H. pylori* reference strain 26695, as demonstrated by the zones of inhibition of 12.3 and 11.3 mm, respectively (Table 2). Two MDR-*H. pylori* isolates, v633 and v1354, which exhibit resistance to metronidazole and clarithromycin, were used in this study. The inhibitory effects against the *H. pylori* strain 26695 of standard antibiotics, metronidazole and clarithromycin alone (without addition of the GMNL-74 and GMNL-185), and two *Lactobacillus* strains (GMNL-229/GMNL-814) with less potent activity as controls were determined as well (Figure S3). As shown in Table 2, GMNL-74 and GMNL-185 demonstrated zones of inhibition ranging from 7.7 to 9.0 mm, indicating potency. These results indicate that GMNL-74 and GMNL-185 possess superior inhibition activity against the growth of both antibiotic-susceptible and MDR-*H. pylori* strains.

### 3.2. Lactobacillus spp. Suppresses H. pylori Adhesion and Invasion Activities

**Figure 1:**
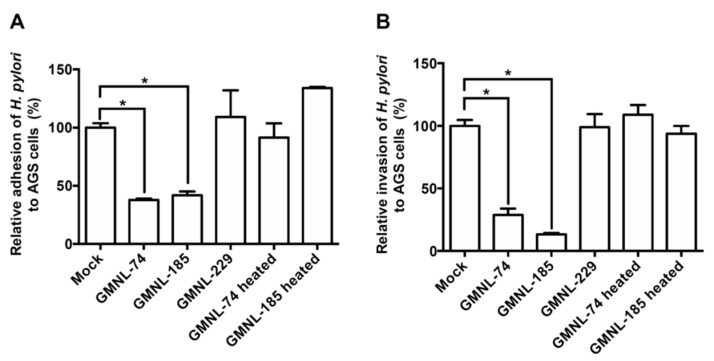
Effects of *Lactobacillus* spp. on *H. pylori* (**A**) adhesion and (**B**) invasion into gastric epithelial cells. AGS cells were treated with live *Lactobacillus* spp. (GMNL-74, 185, and 229) or heat-inactivated *Lactobacillus* spp. (GMNL-74 and 185), followed by infection with *H. pylori* 26695 at multiplicity of infection (MOI) 100 for 6 h. Each experiment result shows the mean ± standard deviation of three independent experiments. * *p* < 0.01.

Adhesion of *H. pylori* to cells is an important initial step to induce pathogenesis in gastric epithelial cells [41]. We therefore investigated whether *Lactobacillus* inhibits the initial step of *H. pylori* adhesion and invasion of AGS cells. As shown in Figure 1A, both GMNL-74 and GMNL-185 exhibited significant anti-adhesion activity against *H. pylori* by 96.7% and 93.2%, respectively, as compared to *H. pylori* infection alone (mock group) (*p* < 0.01). In addition, GMNL-74 and GMNL-185 exhibited dramatic inhibitory activity against *H. pylori* invasion into AGS cells, with a reduction of 99.0% and 99.8% (Figure 1B). However, the control strain, GMNL-229, and heat-inactivated GMNL-74/185 did not exhibit the inhibition of *H. pylori* adhesion and invasion. These results demonstrate that GMNL-74 and GMNL-185 possess anti-adhesion and anti-invasion activities.

### 3.3. Lactobacillus spp. Ameliorates H. pylori-induced Inflammation

**Figure 2:**
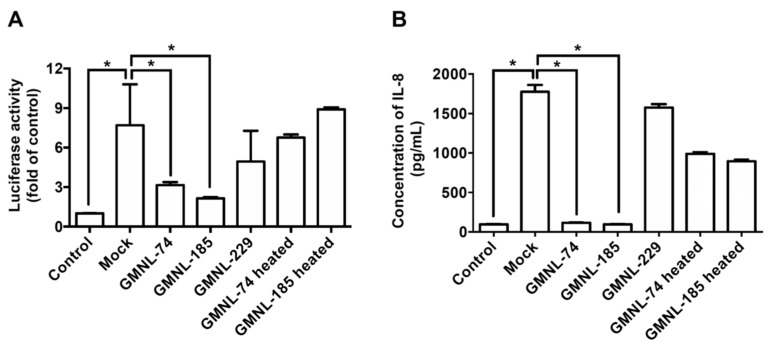
Inhibitory effects of *Lactobacillus* spp. on *H. pylori*-induced inflammation. AGS cells were treated with live *Lactobacillus* spp. (GMNL-74, 185, and 229) or heat-inactivated *Lactobacillus* spp. (GMNL-74 and 185) prior to infection with *H. pylori* 26695 at multiplicity of infection (MOI) 100 for 6 h. The levels of (**A**) nuclear factor-kappa B NF-κB luciferase activity and (**B**) interleukin 8 (IL-8) production were determined as described in the Materials and Methods. Each experiment result shows the mean ± standard deviation of three independent experiments. * *p* < 0.01.

*H. pylori* infection induces NF-κB activation and IL-8 production, which contributes to the inflammation of gastric epithelial cells [8]. We therefore investigated whether *Lactobacillus* inhibits *H. pylori*-induced NF-κB activation. An NF-κB-luciferase reporter [30] was employed to analyze luciferase activity following treatment of the *H. pylori*-infected gastric cells with the *Lactobacillus* strains. Results showed that GMNL-74 and GMNL-185 inhibited luciferase activity by 99.7% and 99.3%, respectively, as compared to cells infected with *H. pylori* infection alone (*p* < 0.01) (Figure 2A). Because *H. pylori* CagA-induced IL-8 expression of cells is mediated via NF-κB activation [8], we further determined *Lactobacillus* inhibition against *H. pylori*-induced IL-8 production. As shown in Figure 2B, IL-8 production was effectively reduced in cells treated with GMNL-74 and GMNL-185 by 97.9% and 88.6%, respectively, when compared with *H. pylori* infection alone (*p* < 0.01). In contrast, neither GMNL-229 nor heat-inactivated GMNL-74/185 attenuated *H. pylori*-induced NF-κB activation and IL-8 production. These results demonstrate that *Lactobacillus* decreased NF-κB activity and IL-8 secretion, which contribute to the amelioration of *H. pylori*-induced inflammation.

### 3.4. Lactobacillus spp. Attenuates H. pylori CagA Translocation and Phosphorylation

**Figure 3:**
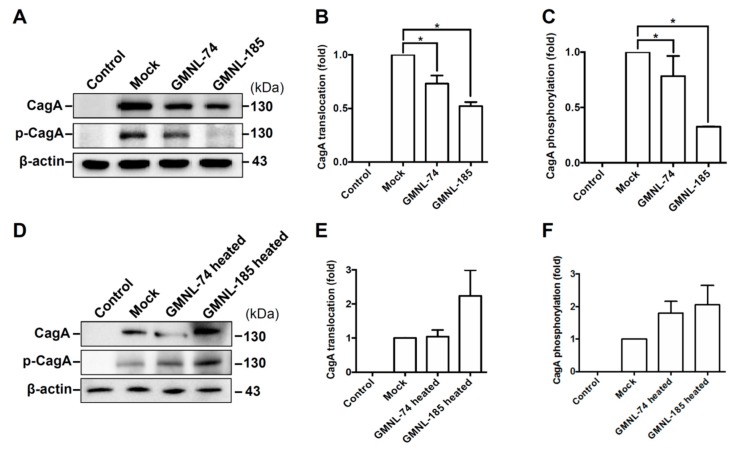
*Lactobacillus* spp. reduced *H. pylori* CagA translocation and phosphorylation. AGS cells were treated with live or heat-inactivated *Lactobacillus* spp. (GMNL-74 and GMNL-185) prior to infection by *H. pylori* 26695 at multiplicity of infection (MOI) 100 for 6 h. (**A**,**D**) Samples were subjected to Western blot analysis. The levels of (**B**,**E**) CagA translocation and (**C**,**F**) CagA phosphorylation were determined by densitometric analysis. Each experiment result shows the mean ± standard deviation of three independent experiments. * *p* < 0.01.

It has been demonstrated that CagA translocation and phosphorylation of *H. pylori* in gastric epithelial cells results in NF-κB activation and IL-8 production, indicating that CagA is crucial for inducing inflammation [8]. We therefore investigated whether *Lactobacillus* attenuated *H. pylori* CagA translocation and phosphorylation in AGS cells. As shown in Figure 3A, the expression levels of CagA translocation and phosphorylation increased remarkably in *H. pylori*-infected cells than those in uninfected cells (control group). However, the levels of translocated and phosphorylated CagA decreased significantly in cells treated GMNL-74 and GMNL-185 compared to cells infected with *H. pylori* alone (mock group). Furthermore, GLMN-74/GMNL-185 both possessed activities that reduced the *H. pylori*-induced cell scattering phenotype and decreased vacuolization in the cytoplasm (Figure S4). In contrast, the heat-inactivated GMNL-74/185 slightly increased CagA translocation and phosphorylation in AGS cells as compared to that infected with *H. pylori* alone (mock group) (Figure 3D). These results indicate that *Lactobacillus* attenuates CagA translocation and phosphorylation, leading to a reduction in the *H. pylori*-induced inflammation of gastric epithelial cells.

### 3.5. Lactobacillus spp. Suppresses Gastric Inflammation in Mice Infected with H. pylori

**Figure 4:**
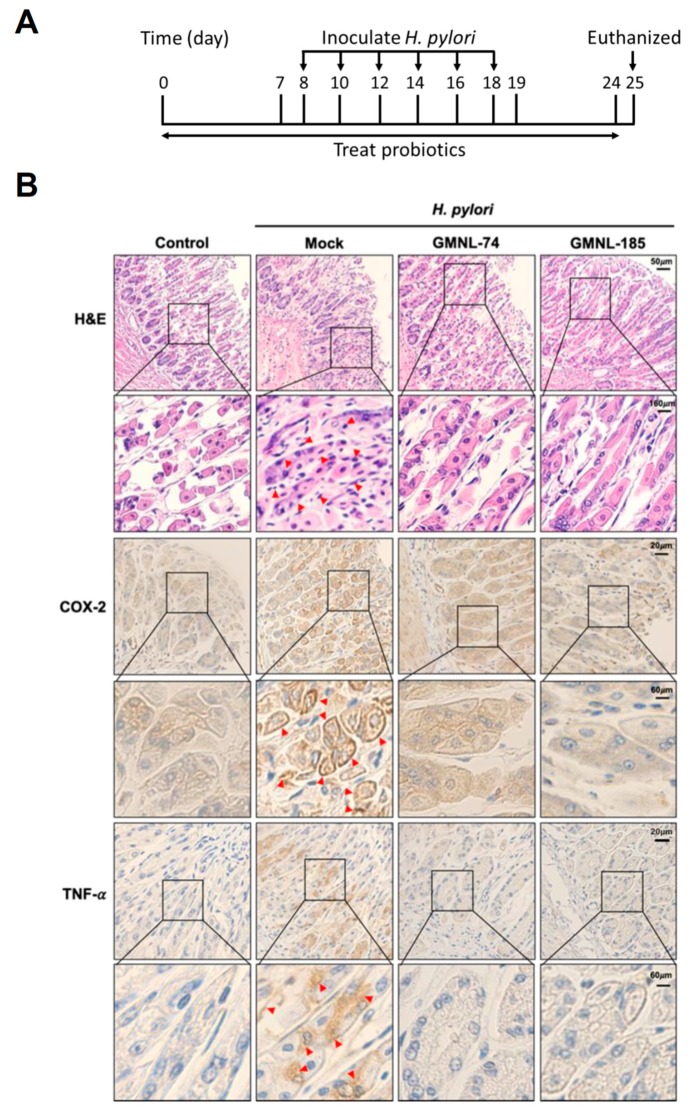
Probiotic *Lactobacillus* spp. alleviated gastric inflammation in mice. (**A**) Mice were fed with *Lactobacillus* spp. (GMNL-74 and GMNL-185) for 24 days followed by intragastric gavage with *H. pylori* 26695 once every 2 days for a total of six administrations. Arrows show the days of *H. pylori* inoculation. (**B**) Mice were euthanized and gastric tissues were subjected to hematoxylin–eosin (H&E) and immunohistochemical (IHC) staining with specific antibodies against cyclooxygenase-2 (COX-2) and tumor necrosis factor (TNF)-α, respectively (original magnification: 200×). The magnified images are shown in the lower panel of each cropped area. Severe infiltration of inflammatory cells in the gastric epithelium (H&E) and pronounced expression of COX-2 and TNF-α in gastric tissues are indicated by red arrows (IHC).

To further investigate whether *Lactobacillus* possesses activity to attenuate *H. pylori*-induced stomach inflammation in vivo, mice were inoculated with the *Lactobacillus* strains followed by infection with *H. pylori* (Figure 4A). After completion of the treatment, mice were euthanized and the gastric tissues were assessed for *H. pylori* infection by the rapid urease test (CLO test) [40]. Our results showed that mice inoculated with *H. pylori* exhibited positive results of the CLO test, while *H. pylori* untreated control mice were negative (Figure S5). Furthermore, mice treated with GMNL-74 or GMNL-185 showed *H. pylori*-negative. The gastric tissues were then analyzed by hematoxylin–eosin (H&E) staining and immunohistochemistry (IHC) assays. As shown in Figure 4B (H&E), the gastric tissue sections from the control group showed a clearly defined epithelium without inflammation. However, the infiltrated leukocytes (arrows in red, Figure 4B H&E) showed in *H. pylori*-infected tissues were more severe than that in the control group. In contrast, *H. pylori*-induced infiltration of inflammatory cells into the gastric epithelium was ameliorated in mice treated with GMNL-74 and GMNL-185. TissueFAXS (TissueGnostics, Tarzana, CA, USA) was then employed to determine the mean intensity of COX-2 and TNF-α expression for IHC staining in gastric tissues (Figure S6). Similar results were observed in the IHC analysis. The results showed that no significant expression of COX-2 and TNF-α (pro-inflammatory cytokines) in the gastric tissues of the control group, whereas a higher expression of these cytokines was seen in gastric tissues infected with *H. pylori* alone (arrows in red, Figure 4B IHC). In contrast, a dramatic decrease of COX-2 and TNF-α was observed in mice fed with GMNL-74 and GMNL-185.

### 3.6. Changes in the Specific Microbiota Members by Treatment with Lactobacillus

**Figure 5:**
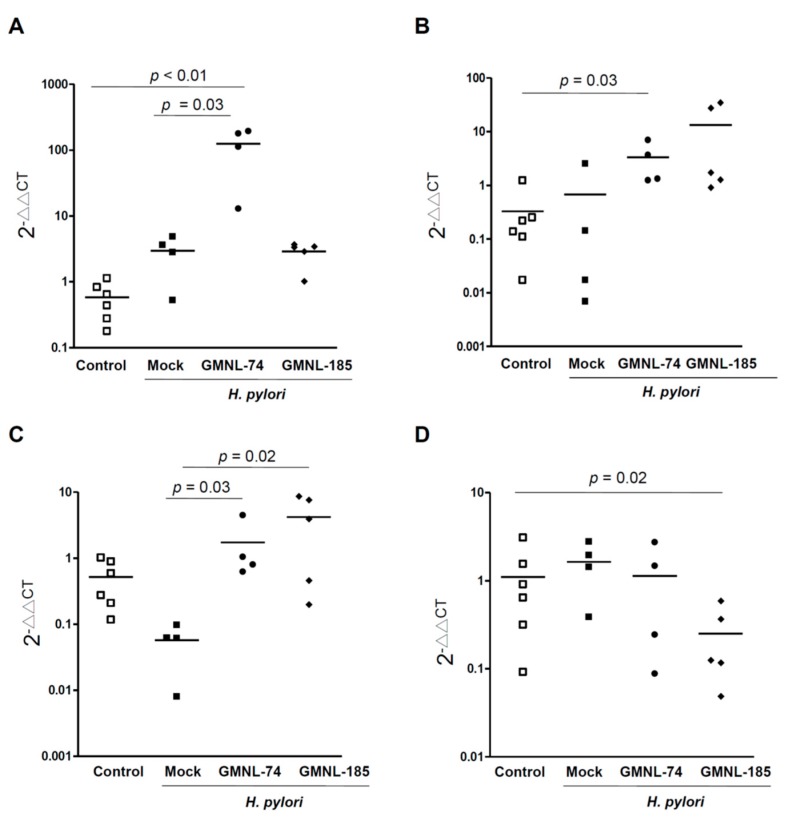
Probiotic *Lactobacillus* spp. altered the gut specific bacteria against *H. pylori* in the mouse stomach. Mouse stool was collected for analyzing qRT-PCR to identify the bacterial species, including (**A**) *Bifidobacterium*, (**B**) *Proteobacteria*, (**C**) *A. muciniphila*, and (**D**) *E. coli*. The alterations of gut specific bacteria were determined as described in the Materials and Methods. Statistical analysis was subjected to the Kruskal-Wallis test with post hoc test by Dunn test. *p* < 0.05 was considered statistically significant.

To further explore whether gut specific bacteria was altered in *H. pylori*-infected mice with *Lactobacillus* treatment, mouse stool was collected. qRT-PCR analysis was performed to identify bacterial phylum (including *Bacteroidetes*, *Actinobacteria*, *Firmicutes*, *Proteobacteria*, *Fusobacteria*), genus (*Bifidobacterium*, *Prevotella*, *Clostridium* cluster I, *Enterococcus*, *Lactobacillus*), and species (*A. muciniphilia*, *S. aureus*, and *E. coli*). As compared to bacterial levels in the controls, an increase in the *Bifidobacterium* and *Proteobacteria*, and a decrease in *A. muciniphilia* were observed in mice infected with *H. pylori* alone (Figure 5). In GMNL-74 fed with *H. pylori*-infected mice, the abundance of *Bifidobacterium*, *Proteobacteria*, and *A. muciniphilia* was increased compared to that in the controls (Figure 5 and Figure S7). In GMNL-185 fed with *H. pylori*-infected mice, there was a significant decrease in the abundance of *Actinobacteria* and *E. coli*, while an increased abundance of *Proteobacteria* and *A. muciniphilia* was also observed. These results indicate that *L. rhamnosus* (GMNL-74) and *L. acidophilus* (GMNL-185) may alter the gut bacterial community and combat *H. pylori* infection.

## 4. Discussion 

*H. pylori* contains a functional CagA that is able to activate NF-κB translocation into the nucleus of gastric epithelial cells [8]. The activation of NF-κB subsequently induces the production of inflammatory mediators, including IL-8, COX-2, and nitric oxide (NO), which are closely associated with tissue inflammation and injury [42]. In this study, we demonstrated that both the probiotics, *L. rhamnosus* (GMNL-74) and *L. acidophilus* (GMNL-185), possess potent activity to inhibit *H. pylori* adhesion to gastric epithelial cells, which in turn, attenuates NF-κB activation and IL-8 production. Our results further showed that these probiotics also ameliorated the expression of *H. pylori*-induced proinflammatory cytokines, TNF-α and COX-2, in mouse gastric epithelia, thus suggesting that they possess anti-inflammatory activity. These findings are consistent with previous studies, which reported that probiotics compete with *H. pylori* adhesion to epithelia, thereby inhibiting the release of inflammatory cytokines, and alleviating gastric inflammation [43]. 

Several virulence factors, including VacA and CagA, are implicated in *H. pylori* carcinogenesis [41]. A persistent stomach infection with *H. pylori* induces secretion of proinflammatory cytokines, including IL-1β, IL-6, IL-8, and TNF-α, which are closely linked to MALT-lymphoma and gastric adenocarcinoma [5]. In addition, *H. pylori* infection alters gastric microbiota, leading to dysbiosis that favors *H. pylori* colonization and gastric cancer development [44]. In the current study, we demonstrated that both GMNL-74 and GMNL-185 inhibited CagA translocation and phosphorylation in gastric epithelial cells, subsequently reducing NF-κB activation and IL-8 production. Furthermore, *Bifidobacterium* spp. and *A. muciniphilia*, which are known to be beneficial, are abundantly present in *H. pylori*-infected mice treated with these probiotics. Elevation of beneficial gut microbiota that decreases *H. pylori* colonization and reduces the detrimental effects of virulence factors, subsequently decreasing the risk of gastric cancer [44]. These lines of evidence indicate that probiotics may be developed as preventive agents for *H. pylori*-induced gastric cancer. 

The gold standard for treating *H. pylori*-infected patients involves a combination of a proton pump inhibitor with several antibiotics [13]. However, failure rates of this treatment increase significantly due to emerging antibiotic resistance, with extensive treatment of *H. pylori* with antibiotics [45]. Noticeably, oral administration of antibiotics, such as amoxicillin, clarithromycin, and metronidazole, alter the balance of microbiota in the gastrointestinal tract [46], including beneficial microbes, such as, *Bifidobacterium* and *Lactobacillus* spp. [47,48], thus adversely affecting the physiological functions of individuals. In contrast, co-administration with probiotics and standard triple-therapy led to increased eradication rates of *H. pylori*, mitigating antibiotic-associated side effects [49]. These findings indicate that probiotics are capable of modulating intestinal microbiota, which further help in eradicating *H. pylori* infection, and decreasing the adverse effects of antibiotics.

We have recently reported that high serum cholesterol levels in humans leads to an increased risk of being infected with *H. pylori* and induction of pathogenesis [35,50]. A population-based case-control study revealed that in patients who were prescribed statins (cholesterol-lowering agents), there was a decrease in *H. pylori* infection, and a reduced risk of peptic ulcer disease and gastric cancer [51,52]. It has been reported that probiotics exhibit anti-obesity effects by lowering serum cholesterol [53,54]. As the administration of mice with GMNL-74 and GMNL-185 ameliorates *H. pylori*-induced inflammation, it is possible that probiotic treatment may lower serum cholesterol to disrupt *H. pylori* infectivity in this case. It remains to be investigated whether cholesterol levels are affected favorably by probiotics, and whether this modulation contributes to the alleviation of *H. pylori*-induced inflammation.

In this study, we chose four bacterial species that represent the major population of gut microbiota that may be altered by treatment with the probiotics. Our results showed an increase in *Bifidobacterium* and *A. muciniphilia*, and a decrease in *E. coli* and *Clostridium* cluster I when *H. pylori*-infected mice were treated with probiotics. This evidence indicates that probiotics are effective in protecting mice infected with *H. pylori* by changing the specific microbiota members. However, the composition of indigenous microbiota is very complicated and is known to regulate the pathophysiology, immunity, and metabolism of hosts. Therefore, long-term systemic studies and extensive exploration of gut microbiota along with their ecosystems are warranted.

## 5. Conclusions

Our results showed that two *Lactobacillus* spp., GMNL-74 and GMNL-185, contain potent antimicrobial activity against *H. pylori* growth and inflammation. The results from this study demonstrate that both the probiotics, GMNL-74 and GMNL-185, could be developed as preventive agents for inhibition of *H. pylori* infection and alleviation of inflammation.

## Figures and Tables

**Table 1:** Primers used for the quantification of gut specific bacteria.

Bacterial Species	Primer	Nucleotide Sequence (5’-3’)
Total bacteria	Forward	GTGSTGCAYGGYTGTCGTCA
	Reverse	ACGTCRTCCMCACCTTCCTC
*Bifidobacterium*	Forward	CGCGTCYGGTGTGAAAG
	Reverse	CCCCACATCCAGCATCCA
*Akkermansia muciniphila*	Forward	CAGCACGTGAAGGTGGGGAC
	Reverse	CCTTGCGGTTGGCTTCAGAT
*Escherichia coli*	Forward	CATGCCGCGTGTATGAAGAA
	Reverse	CGGGTAACGTCAATGAGCAAA
*Clostridium* cluster I	Forward	TACCHRAGGAGGAAGCCAC
	Reverse	GTTCTTCCTAATCTCTACGCAT
*Bacteroides-Provetella*	Forward	AAGGTCCCCCACATTGG
	Reverse	CCGCGGCKGCTGGCAC
*Proteobacteria*	Forward	TGGTGTAGGGGTAAAATCCG
	Reverse	AGGTAAGGTTCTTCGYGTATC
*Actinobacteria*	Forward	TGTAGCGGTGGAATGCGC
	Reverse	AATTAAGCCACATGCTCCGCT
*Fusobacteria*	Forward	AAGCGCGTCTAGGTGGTTATGT
	Reverse	TGTAGTTCCGCTTACCTCTCCAG
*Enterococcus*	Forward	CCCTTATTGTTAGTTGCCATCATT
	Reverse	ACTCGTTGTACTTCCCATTGT
*Firmicutes*	Forward	GCGTGAGTGAAGAAGT
	Reverse	CTACGCTCCCTTTACAC
*Bacteroidetes*	Forward	CTGAACCAGCCAAGTAGCG
	Reverse	CCGCAAACTTTCACAACTGACTTA

**Table 2:** Inhibitory effect of probiotic *Lactobacillus* spp. on *H. pylori* 26695 (ATCC 700392) and multidrug-resistant (MDR) isolates v633 and v1354.

	Inhibition Zone (mm) ^‡^		
	Reference Strain	MDR-*H. pylori* ^†^	
Treatment	26695	v633	v1354
GMNL-74	12.3 ± 0.5	8.3 ± 0.5	8.7 ± 0.5
GMNL-185	11.3 ± 0.5	7.7 ± 0.5	9.0 ± 0.8
GMNL-229	0	0	0
GMNL-814	0	0	0
MRS broth	0	0	0
Clarithromycin	21.5 ± 0.3	0	0
Metronidazole	18.3 ± 0.4	0	0

^†^ Strains v633 and v1354 were multidrug resistant (MDR) clinical isolates, which showed resistant to clarithromycin and metronidazole [32]. Standard antibiotics, clarithromycin (50 μg/mL) and metronidazole (800 μg/mL), were used as controls. *H. pylori* showed without inhibition zone. Results were shown as the mean of different analysis of three independent experiments. ^‡^ Data were shown as means ± SD.

## Supplementary Materials

The following are available online at http://www.mdpi.com/2077-0383/8/1/90/s1, Figure S1: Flowchart of the screening process and experimental procedure. Figure S2: Identification of GMNL-74 and GMNL-185. (A) Represents partial 16S rDNA sequences of *Lactobacillus* strains GMNL-74 (upper panel) and GMNL-185 (lower panel). (B) Represents RAPD profiles of GMNL-74 and GMNL-185, Figure S3: Agar disk diffusion method to analyze the inhibition activity of standard antibiotics and *Lactobacillus* strains on *H. pylori* 26695 strain, Figure S4: Hummingbird phenotype and vacuolization assay, Figure S5: CLO test for analyzing *H. pylori* infection in mouse stomach, Figure S6: The mean intensity of COX-2 and TNF-α expression for IHC staining in gastric tissues was determined using TissueFAXS. The percentage of cells positive for staining were calculated and shown in the upper right side of each sample, Figure S7: qRT-PCR analysis was performed to identify bacterial phylum: (A) *Bacteroidetes*, (B) *Actinobacteria*, (C) *Firmicutes*, (D) *Prevotella*, (E) *Fusobacteria*, and genus (F) *Enterococcus*, (G) *Lactobacillus,* (H) *Clostridium* cluster I. Table S1: Fermentation of carbohydrates was determined using API 50 CHL to identify *Lactobacillus* and related genera.
