# Supplementary material for: Probiotic Lactobacillus spp. Act Against Helicobacter pylori-induced Inflammation
Source: J Clin Med. 2019 Jan 14;8(1):90. doi: 10.3390/jcm8010090 (PMC6352136; doi:10.3390/jcm8010090)
Supplement: Supplementary file 1 [file jcm-08-00090-s001.zip › Supplementary files/Figures S1-S7.pdf]

Fig. S1

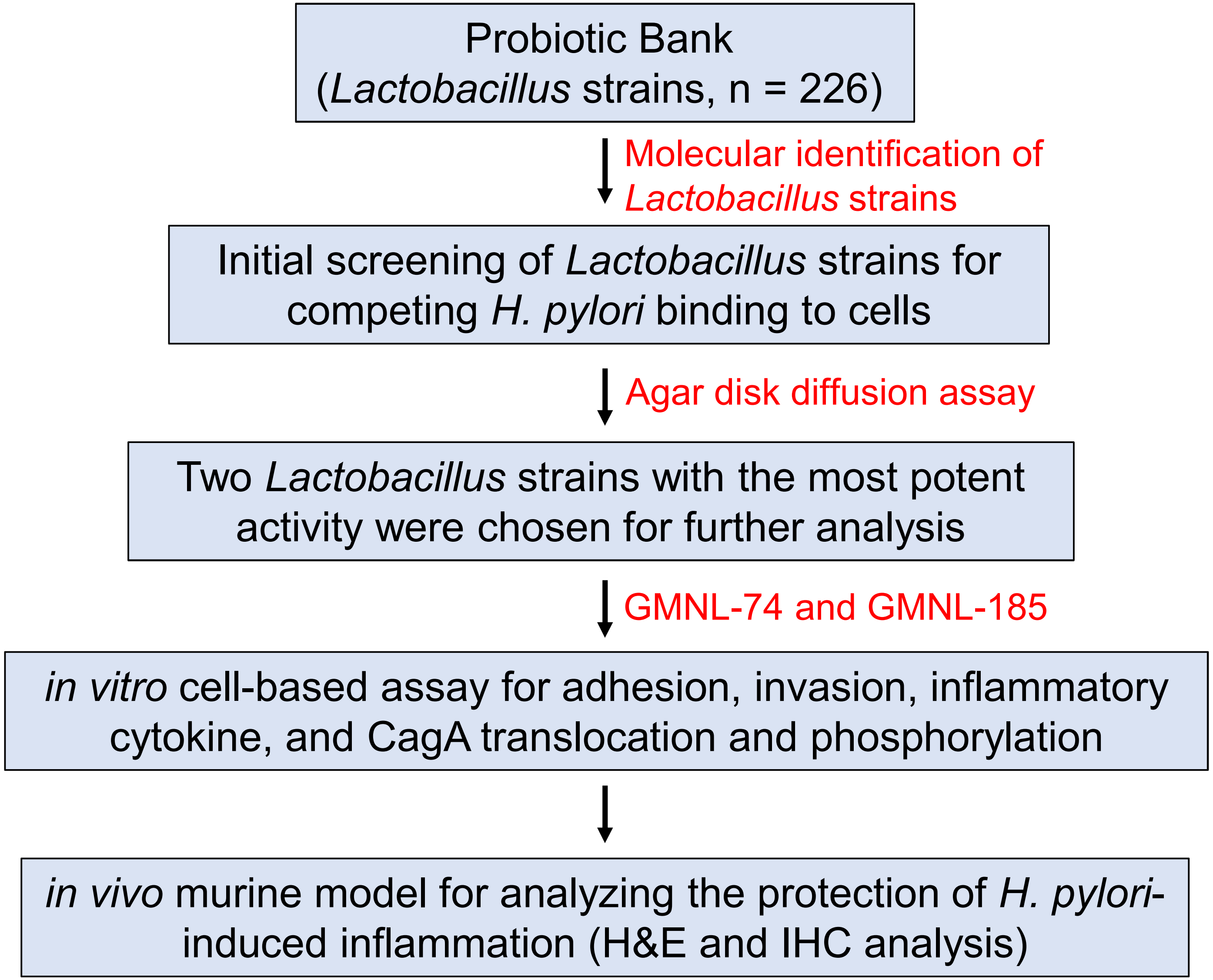
Probiotic Bank
(*Lactobacillus* strains, n = 226)
Molecular identification of *Lactobacillus* strains
Initial screening of *Lactobacillus* strains for competing *H. pylori* binding to cells
Agar disk diffusion assay
Two *Lactobacillus* strains with the most potent activity were chosen for further analysis
GMNL-74 and GMNL-185
in vitro cell-based assay for adhesion, invasion, inflammatory cytokine, and CagA translocation and phosphorylation
in vivo murine model for analyzing the protection of *H. pylori*-induced inflammation (H&E and IHC analysis)

Fig. S2

A

GMNL-74

AATGCGCGTGCTATACATGCAAGTCGAACGAGTTCTGATTATTGAAAG
GTGCTTGCATCTTGATTTAATTTTGAACGAGTGGCGGACGGGTGAGTAA
CACGTGGGTAACCTGCCCTTAAGTGGGGGATAACATTTGGAAACAGAT
GCTAATACCGCATAAATCCAAGAACCGCATGGTTCTTGGCTGAAAGAT
GGCGTAAGCTATCGCTTTTGGATGGACCCGCGGCGTATTAGCTAGTTGG
TGAGGTAACGGCTCACCAAGGCAATGATACGTAGCCGAACTGAGAGGT
TGATCGGCCACATTGGGACTGAGACACGGCCCAAACTCCTACGGGAGG
CAGCAGTAGGGAATCTTCCACAATGGACGCAAGTCTGATGGAGCAACG
CCGCGTGAGTGAAGAAGGCTTTCGGGTCGTAAAACTCTGTTGTTGGAG
AAGAATGGTCGGCAGAGTAACTGTTGTCGGCGTGACGGTATCCAACCA
GAAAGCCACGGCTAACTACGTGCCAGCAGCCGGGG

GMNL-185

CTGCGGTGCTATACATGCAAGTCGAGCGAGCTGAACCAACAGATTCAC
TTCGGTGATGACGTTGGGAACGCGAGCGGCGGATGGGTGAGTAACACG
TGGGGAACCTGCCCCATAGTCTGGGATACCACTTGGAAACAGGTGCTA
ATACCGGATAAGAAAGCAGATCGCATGATCAGCTTATAAAAGGCGGCG
TAAGCTGTCGCTATGGGATGGCCCCGCGGTGCATTAGCTAGTTGGTAGG
GTAACGGCCTACCAAGGCAATGATGCATAGCCGAGTTGAGAGACTGAT
CGGCCACATTGGGACTGAGACACGGCCCAAACTCCTACGGGAGGCAGC
AGTAGGGAATCTTCCACAATGGACGAAAGTCTGATGGAGCAACGCCGC
GTGAGTGAAGAAGGTTTTCGGATCGTAAAGCTCTGTTGTTGGTGAAGA
AGGATAGAGGTAGTAACTGGCCTTTATTTGACGGTAATCAACCAGAAA
GTCACGGCTAACTACGTGCCAGACG

B

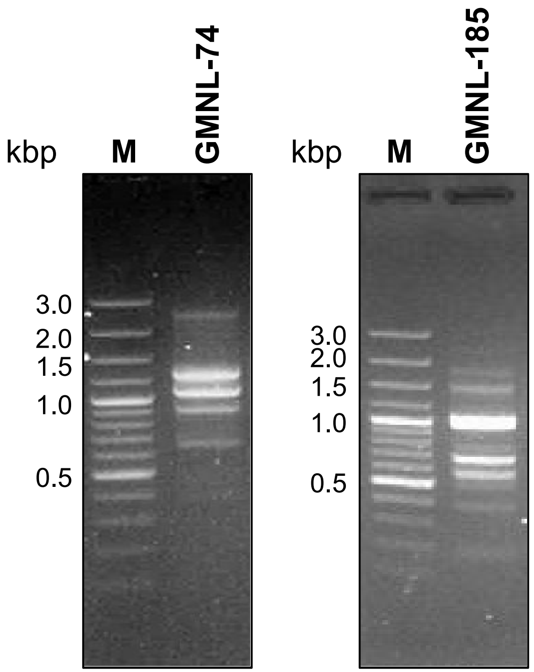

Fig. S3

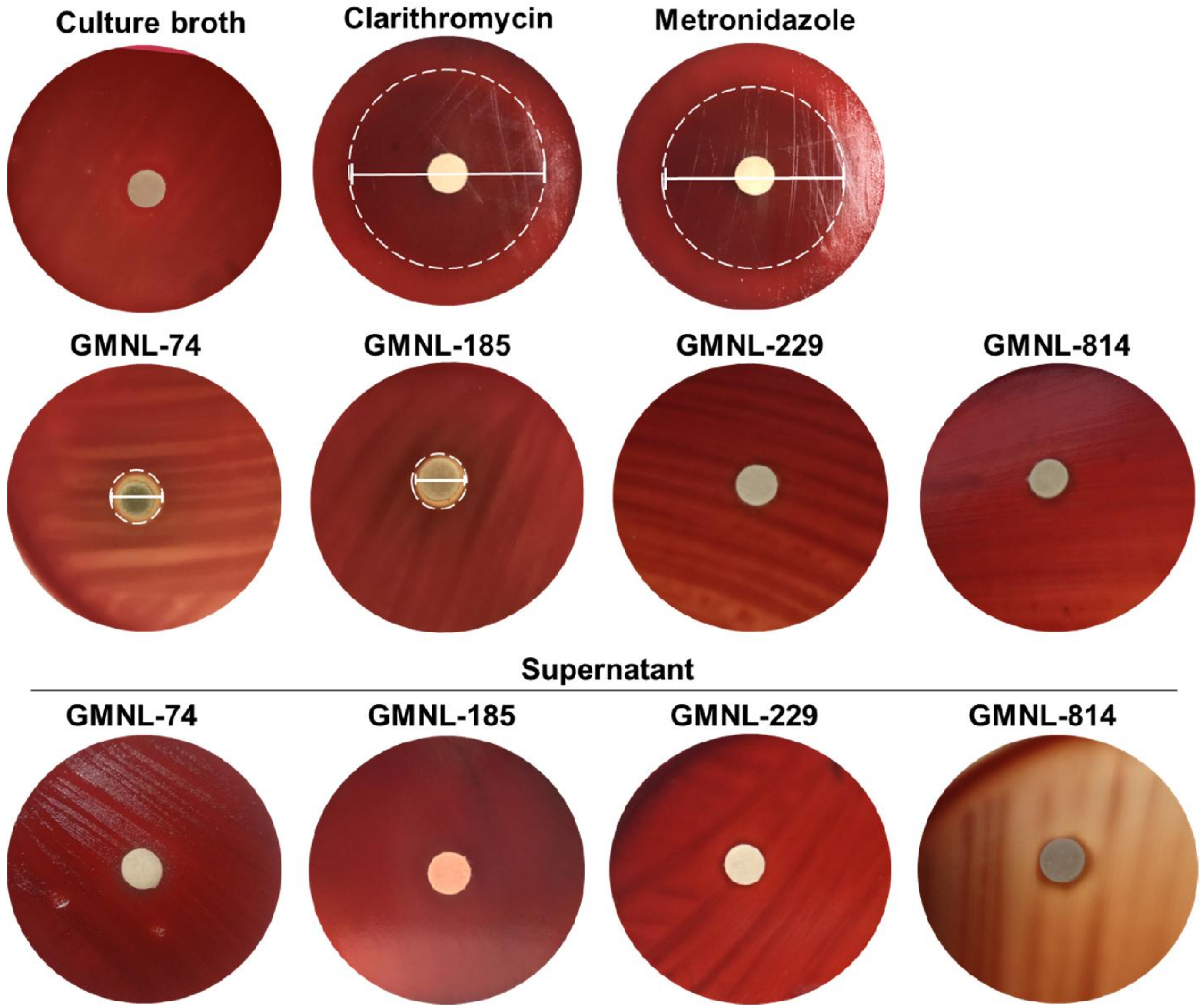

Culture broth
Clarithromycin
Metronidazole
GMNL-74
GMNL-185
GMNL-229
GMNL-814
Supernatant
GMNL-74
GMNL-185
GMNL-229
GMNL-814

Fig. S4

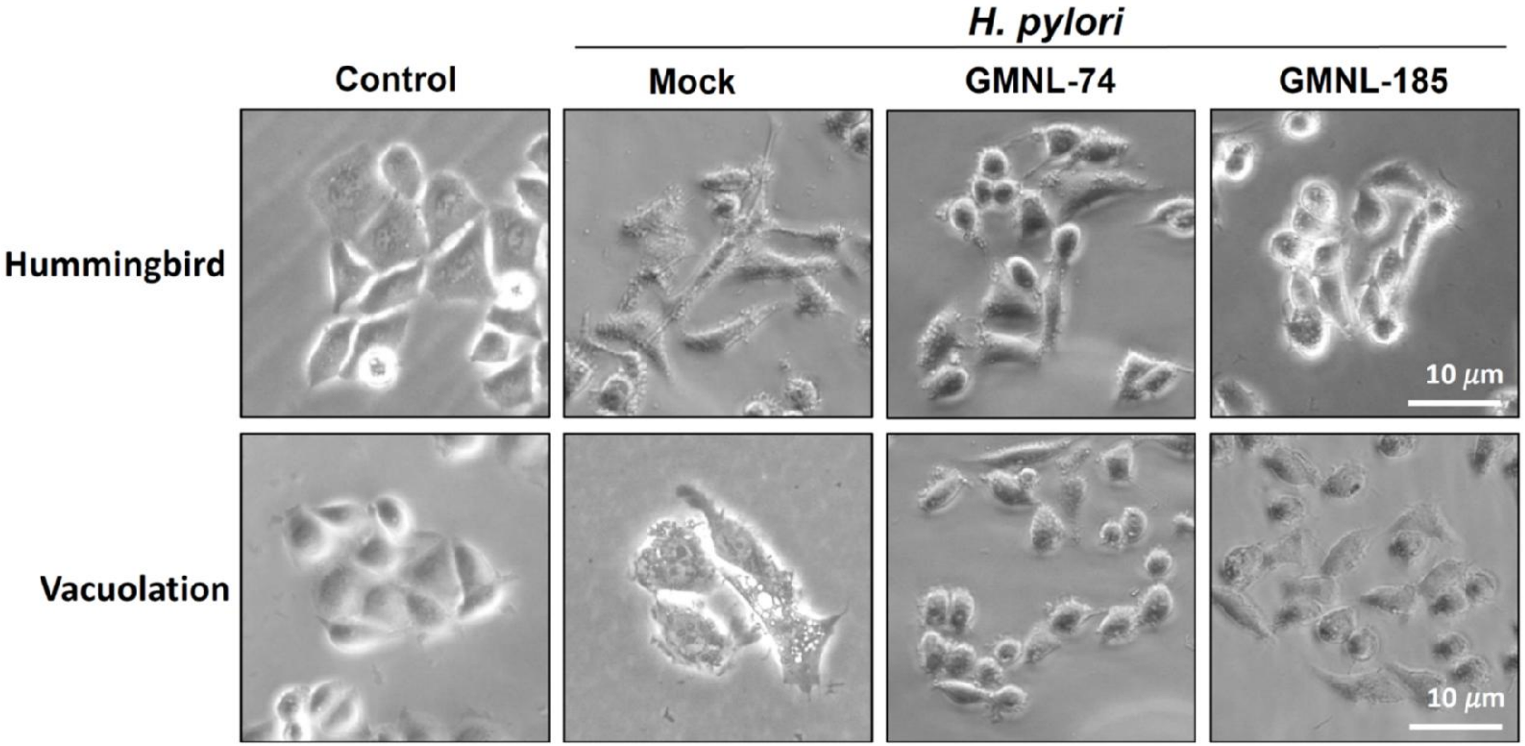
H. pylori
Control
Mock
GMNL-74
GMNL-185
Hummingbird
Vacuolation
10 μm
10 μm

Fig. S5

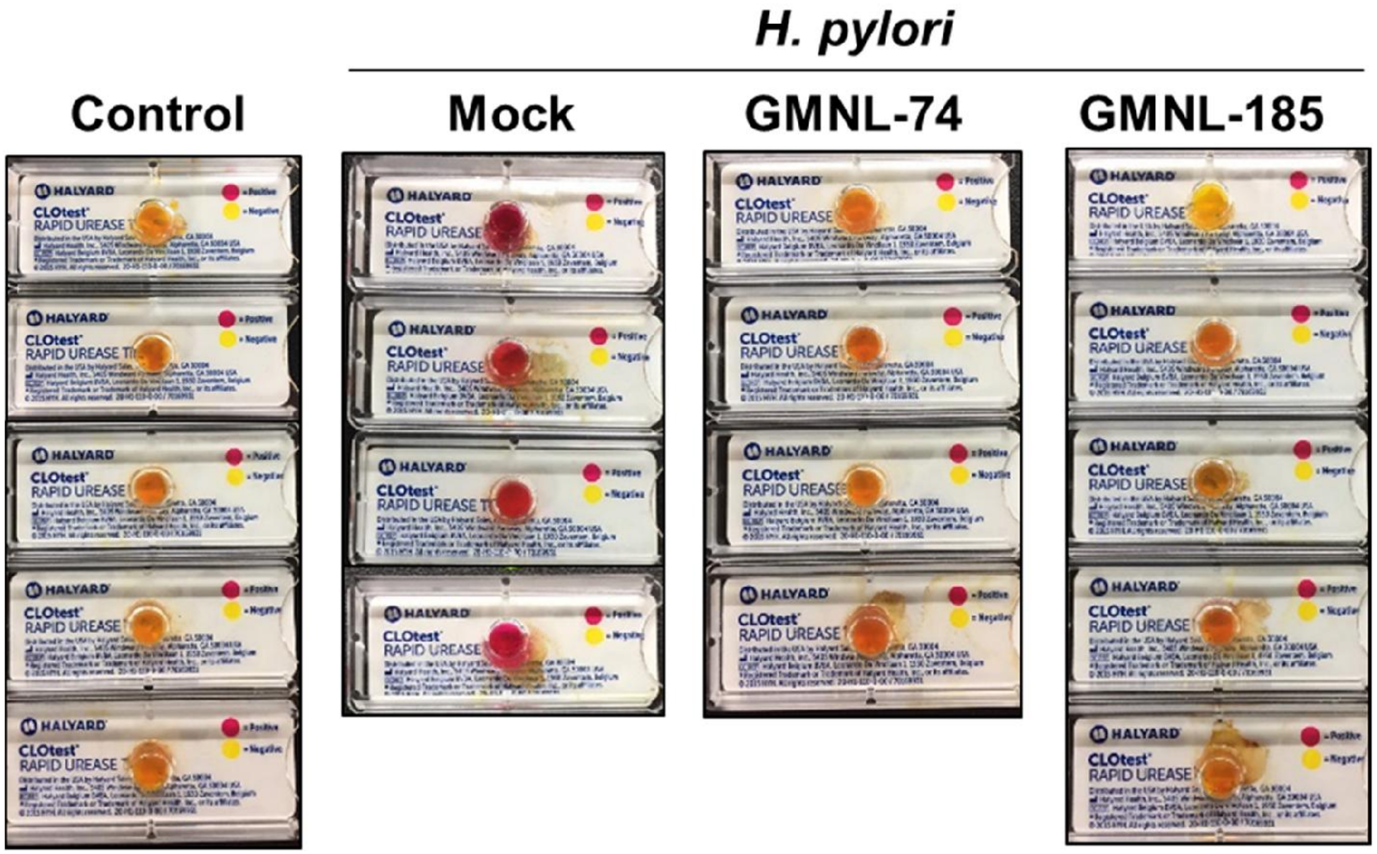

H. pylori
Control
Mock
GMNL-74
GMNL-185
HALYARD
CLOtest
RAPID UREASE

Fig. S6

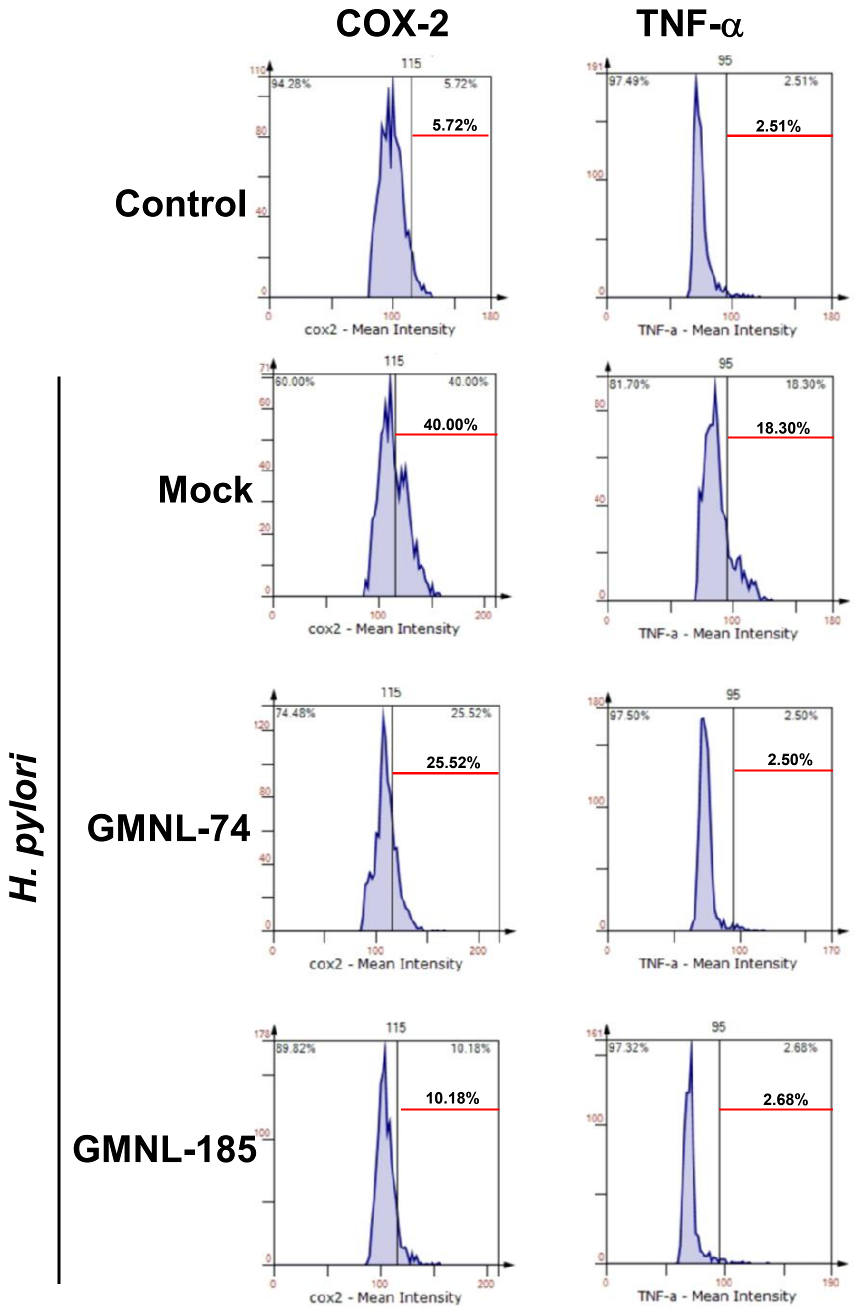

COX-2
TNF-α
Control
H. pylori
Mock
GMNL-74
GMNL-185
5.72%
2.51%
40.00%
18.30%
25.52%
2.50%
10.18%
2.68%
cox2 - Mean Intensity
TNF-a - Mean Intensity

Fig. S7

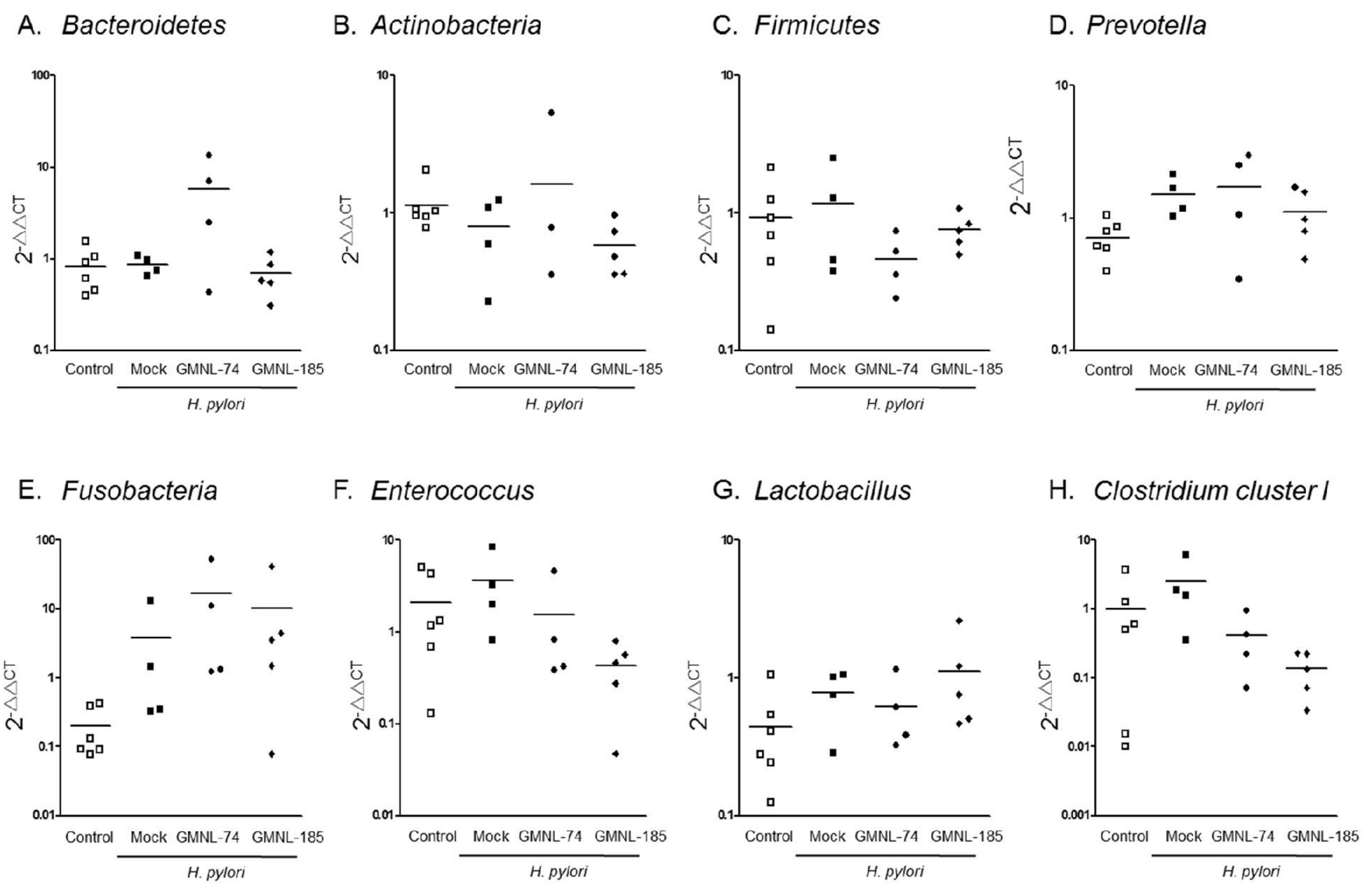

A. Bacteroidetes
B. Actinobacteria
C. Firmicutes
D. Prevotella
E. Fusobacteria
F. Enterococcus
G. Lactobacillus
H. Clostridium cluster I
2-△△CT
Control
Mock
GMNL-74
GMNL-185
H. pylori
