# Supplementary material for: Probiotic Lactobacillus spp. Act Against Helicobacter pylori-induced Inflammation
Source: J Clin Med. 2019 Jan 14;8(1):90. doi: 10.3390/jcm8010090 (PMC6352136; doi:10.3390/jcm8010090)
Supplement: Supplementary file 1 [file jcm-08-00090-s001.zip › Supplementary files/Table S1.docx]

**Table S1.** Fermentation of carbohydrates was determined using API 50 CHL to identify *Lactobacillus* and related genera

| Strips no. | Carbohydrates substrate | GMNL-74 | GMNL-185 | Strips no. | Carbohydrates substrate | GMNL-74 | GMNL-185 | Strips no. | Carbohydrates substrate | GMNL-74 | GMNL-185 |
| --- | --- | --- | --- | --- | --- | --- | --- | --- | --- | --- | --- |
| 1 | Glycerol | - | - | 18 | D-Mannitol | + | - | 35 | D-Raffinose | - | + |
| 2 | Erythritol | - | - | 19 | D-Sorbitol | + | - | 36 | Amidon (starch) | - | - |
| 3 | D-Arabinose | - | - | 20 | Methyl-α-D-mannopyranoside | - | - | 37 | Glycogen | - | - |
| 4 | L-Arabinose | - | - | 21 | Methyl-α-D-glucopyranoside | + | - | 38 | Xylitol | - | - |
| 5 | D-Ribose | + | - | 22 | N-Acetyl glucosamine | + | + | 39 | Gentiobiose | + | + |
| 6 | D-Xylose | - | - | 23 | Amygdalin | + | + | 40 | D-Turanose | + | - |
| 7 | L-Xylose | - | - | 24 | Arbutin | + | - | 41 | D-Lyxose | - | - |
| 8 | D-Adonitol | - | - | 25 | Esculin ferric citrate | + | + | 42 | D-Tagatose | + | - |
| 9 | Methyl-β-D-Xylopyranoside | - | - | 26 | Salicin | + | + | 43 | D-Fucose | - | - |
| 10 | D-Galactose | + | + | 27 | D-Cellobiose | + | + | 44 | L-Fucose | - | - |
| 11 | D-Glucose | + | + | 28 | D-Maltose | - | + | 45 | D-Arabitol | - | - |
| 12 | D-Fructose | + | + | 29 | D-Lactose (bovine origin) | + | + | 46 | L-Arabitol | - | - |
| 13 | D-Mannose | + | + | 30 | D-Melibiose | - | - | 47 | Potassium gluconate | ? | - |
| 14 | L-Sorbose | + | - | 31 | D-Saccharose (sucrose) | - | + | 48 | Potassium 2-ketogluconate | - | - |
| 15 | L-Rhamnose | + | - | 32 | D-Trehalose | + | + | 49 | Potassium 5-ketogluconate | - | - |
| 16 | Dulcitol | - | - | 33 | Inulin | - | - |  |  |  |  |
| 17 | Inositol | - | - | 34 | D-Melezitose | + | - |  |  |  |  |
